# Design and Implementation of a Low-Cost Perception System for Aerial Robots in Confined Spaces

**DOI:** 10.3390/s26041140

**Published:** 2026-02-10

**Authors:** Susan Basnet, Jens Christian Andersen, Evangelos Boukas

**Affiliations:** Department of Electrical and Photonics Engineering, Technical University of Denmark, 2800 Kongens Lyngby, Denmark; jcan@dtu.dk (J.C.A.); evanb@dtu.dk (E.B.)

**Keywords:** ToF sensors, unmanned aerial vehicle (UAVs), confined spaces, collision avoidance, autonomous robots

## Abstract

Operating an aerial vehicle in a confined space, such as a vessel ballast tank, is a major challenge in terms of localization, perception, and control due to limited visibility, constrained maneuvering space, and the absence of reliable (if any) GNSS signals. This paper addresses the design considerations for a quadcopter in confined spaces, focusing on a novel perception system using 12 VL53L8CX time-of-flight (ToF) sensors from STMicroelectronics. These sensors are used for enhanced perception and collision avoidance while flying in confined spaces, making them a suitable alternative to bulky LiDAR systems, reducing weight, cost, and required computational power. These sensors are placed strategically around the quadcopter to cover 360° radial view within a 4 m range. Experiments are conducted to test the reliability and repeatability of the integrated system, along with its synchronization feature. Furthermore, the applicability is verified by flying in confined and cluttered spaces, both in simulation and the real world. This design and study aims to establish a baseline for lightweight, compact, and safe navigation for small drones in confined and featureless environments.

## 1. Introduction and Motivation

The use of unmanned aerial vehicles (UAVs)—or aerial robots—offers significant advantages in inspection and exploration, particularly in areas where human life is at risk. Minimizing human involvement to increase efficiency, especially when performing repetitive tasks such as inspection of confined spaces, e.g., in ballast tanks or cargo holds of large vessels, using a UAV is very beneficial. This kind of work is also considered dangerous, dirty, and dull because every week on average one person is killed while working in these kinds of enclosed spaces [1]. In these areas, the applicability of ground robots is limited due to the presence of numerous complex structures, passages, and openings. Hence, considerable research has been conducted on aerial vehicles for applications in such environments. Although they could perform better than ground robots, aerial robots face their own challenges, including dealing with turbulence, maneuverability constraints, magnetic interference from metallic enclosures, and operating in a GNSS-denied environment to ensure safe and collision-free flight [2,3,4,5]. For this reason, the selection of appropriate sensors is very critical during the design phase of a quadcopter. Furthermore, there exists payload and total weight constrains when flying small and mid-sized aerial vehicles, which are ideal for confined space inspection [6,7,8]. There are many UAVs designed to fly in confined and cluttered spaces, utilizing a combination a variety of visual [9,10,11] or LiDAR [12,13] sensors for perception and localization. However, due to the limitations in visibility, power efficiency, and weight, they are not an ideal solution for small UAVs in a ballast tank. Thus, the use of lightweight time-of-flight sensors for perception can serve a good purpose in such environments without affecting the flight time of the UAV.

### 1.1. Quadcopter Design with Ducted Propellers

Since the early days of UAVs, there has been active research on developing a system to achieve good aerodynamic performance in both horizontal and vertical flight at low speeds [14]. Initially, studies mainly focused on flow patterns for aircrafts capable of performing vertical takeoff and landing for different velocities [15,16]. However, recently, the focus has shifted towards studying multi-copters and bounding aerodynamic effects on them [17,18]. Ref. [19] quantifies the ground effect for both single- and multirotor systems. However, these were addressed through a control strategy rather than a design parameter. The consequences of the ceiling effect were studied, and a design modification was made to increase efficiency in [20]. Furthermore, ref. [21] investigated duct shape optimization considering its interference with a quadcopter in hover using different airfoils and assessed it through flow simulation. A duct for the multirotor was designed in [22] using the momentum theory, and experiments were conducted to assess the overall effect of proximity in terms of thrust, power, and flight time. Taking it a step further, ref. [23] performed a numerical simulation for coaxial propellers in ducts. However, these approaches were inefficient in terms of thrust generation and power consumption with respect to a general ducted fan.

### 1.2. Work on Perception System

There has always been a challenge in incorporating a reliable and low-cost perception system for UAVs flying autonomously in confined GNSS-denied environments. There are various criteria for the selection of onboard sensors based on the application of the UAV. The reliability and robustness of that sensor are the major factors dictating the success of any mission regardless of the platform used. Hence, selecting the right sensor can be a daunting task as it provides the foundation for the choice of various navigation, localization, motion planning, or obstacle avoidance algorithms. To address this, numerous studies have been conducted using various approaches to calculate the position or velocity of the UAV. Among those, light detection and ranging (LiDAR) [24,25,26], radio detection and ranging (RADAR) [27,28], Ultrasonic [29,30], and vision [31,32,33] based approaches are the prominent ones. However, they have their own limitations considering the payload, power consumption, computational load, and confined dark spaces. For example, even LiDAR sensors, theoretically ideal for the perception and localization in ballast tanks, fall short for a low-cost UAV with less payload and computation capacity, as they provide half a million 3D pointcloud data within a few seconds [34,35]. Another option is using LiDAR rangefinders [36,37], which use a single-beam laser to measure the depth to a single point. However, they are mostly used for above-ground level (AGL) ranging due to their single target property. Furthermore, 1D LiDAR [38,39] are lightweight and have a reasonable range for confined spaces, but they fall short in the field of view. Although single range finders [40,41] like 1D LiDAR are cost-effective and lightweight, they are not robust and have limited sensing capabilities for a mid-size UAV. On the other hand, 2D LiDARs [42,43] only have plane coverage, making them susceptible to protruding obstacles. Additionally, RADAR could also be a good alternative, with the implementation of additional methods, such as those in [44], to eliminate outliers and aid in correcting the drift in IMU using recursive Random Sample Consensus (RANSAC). Again, this method also falls short in terms of weight, size, power consumption, and cost [34]. Ultrasonic rangefinders [45], often characterized by sufficient range, frequency, and accuracy, could be an option for confined spaces only if the former were not susceptible to temperature, pressure, and humidity. They are also highly susceptible to echoes and false reflections, particularly when operating within metallic containers, such as ballast tanks. There has been extensive research on fusing complementary sensors to enhance robustness and accuracy. The fusion of different modalities of sensors like IMU and barometers with a complementary filter [46], IMU, ultrasonic, and barometer fusion [47], IMU, barometer, and laser rangefinder with an extended Kalman filter [48] are used for better estimation. Although these techniques are efficient in state estimation on the UAV, they cannot give a complete safety boundary while operating in confined and cluttered spaces.

Moreover, whether using monocular, stereo, or multiple cameras, the performance of a vision-based system regarding perception and localization is highly dependent on the light conditions and the features of the surroundings. Although such systems are popular for their low weight and power consumption attributes, they also fall short in terms of motion blurring and require the consumption of relatively high computational resources. There are some methods that operate on features [49] for sparsity with robustness against rolling shutter artifacts and some that directly operate on image pixel intensities [50] for performance against illumination variations. Semi-direct methods leverage a combination of each of these traits [51]. However, the computational cost for real-time applications remains a significant concern for all these vision methods. Similarly, due to the limitations of the field of view and operating constraints with static obstacles, IR-based cameras [52] and event-based cameras [53] are not ideal choices. LiDAR-based perception has come up as an advantageous one, especially in confined spaces due to its low sensitivity to light, negligible dependency on environmental conditions, and ability to detect multiple obstacles simultaneously with robust depth perception [54]. However, these LiDAR systems are quite power-hungry, and some are computationally expensive, which makes them unsuitable for simple and effective perception in navigation. For example, some of the commonly used LiDARs in aerial robotics are Velodyne (8 W) [24], Hokuyo UST-10LX (2.94 W) [55], and Ouster OS1-16 (16 W) [56]. Hence, maximizing flight time by optimizing the sparsity of perception is the major challenge in modern autonomous flights. Recent studies have investigated multi-zone ToF sensors, particularly the VL53L5CX family [57,58]. These devices have demonstrated promising results in accuracy, pixel-level error, and usable range across varying illumination conditions. However, existing implementations are typically limited to four sensors connected via a single custom interface board on the Crazyflie platform. Scaling this approach to larger drones with longer wiring introduces additional challenges, including increased susceptibility to interference and noise, signal attenuation, reduced coverage, and greater hardware complexity. Other work has explored expanding to larger sensor arrays, such as integrating tactile sensors on flexible PCBs wrapped around robotic arms for human–robot interaction [59,60]. Although these systems show that high sensor counts are feasible, they generally lack synchronized sampling and depend on relatively large physical installations (on the order of 80 cm), making them unsuitable for our target application. There still lacks a proper balance in cost, weight, modularity, robustness, and efficiency in the perception system, especially for small-sized aerial robots operating in dark and confined spaces. The use of small ToF sensors can have better prospects, but lacks the degree of coverage in small to big robots, making scalability and adaptation more important. Apart from that, it should have real-time perception capability for agile flight in tight surroundings. This brings us to the core concept of this study, where we design a novel system, extending the work from [61] to maximize the ability to perceive for drones larger than nano platforms using lightweight, low-cost time-of-flight sensors.

Considering the research gaps based on the objective of our application, this paper aims to describe the work involved in designing a UAV optimally for flying in confined spaces and the perception system that could ideally fit these flying areas. Recent works focusing on the design of UAVs and active concepts used for perception in confined spaces are discussed in Section 1. Section 2 is dedicated to describing the architecture of the whole system. It highlights the platform components, including the likely airframe, materials, electronics, and sensors used. Section 3 describes the software-in-the-loop architecture with the frame transformations needed for traversing sensor readings into the actual application. Following this, Section 4 presents the utilization of the perception system for collision avoidance and prevention in both simulation and real experiments. The experiments demonstrate the effectiveness of the design, and the obtained results are discussed concurrently. Section 5, finally provides the concluding remarks and some future endeavors. This paper contributes to the following major areas:

- **Unique UAV Design**: A novel UAV design and system architecture is proposed to tackle the problems encountered flying in a confined space optimally.
- **Perception System**: A unique system of 12 time-of-flight sensors (VL53L8CX) is designed for active perception around the UAV.

## 2. System Design

In this section, the design considerations and the selection of the system components used are discussed. The platform is built in a modular fashion, where each structure has its unique functionality. Additionally, the section explains the motivation behind the design and sensor choices, which directly affect the UAV’s maneuverability, endurance, and ability to operate safely in confined spaces.

### 2.1. Airframe

A fundamental component of this paper is the structural design of the quadcopter, with a focus on its application in confined spaces. Some of the basic, yet crucial, factors that need to be considered include compact size and form factor, collision tolerance, modularity, and agile flight while flying in a ballast tanks or cargo holds. Bearing this in mind, the quadcopter is designed to consist of two major structural parts: the internal structure and the ducts. All the cameras and the companion computer (Holybro Jetson Pixhawk baseboard) are attached to the internal structure. In contrast, the ducts protect the propellers, act as a landing gear, house 10 VL53L8CX sensors, and 4 LED strips. A 3 mm thick carbon fiber material of density $0.001152\;{\mathrm{gm}/\mathrm{mm}}^{3}$ is used in all structural supports, including the upper plate where the motors are held, the lower plate where the companion computer is located, and the support spacer between these two plates. Similarly, the ducts are printed with the selective laser sintering (SLS) method with Nylon “high-performance polyamide 12” material (HP-PA-12) of density 1.01 g/cm^3^. This provides an overall structure that offers both lightweight and high-impact resistance properties. Furthermore, holders for the Raspberry Pi camera module are also of the same material, contributing to a total airframe weight of 303 g.

The propeller plane is tilted by 5° towards the center of the quadcopter to increase the platform’s maneuverability through thrust vectoring [62]. It has been proven that the tilted propeller plane helps stabilize the vehicle during hover and enhances the overall controllability of the quadcopter [63]. Moreover, the flight stability is improved by a factor of approximately 1.3 when compared to no tilt configuration, calculated through the square of the error (SSE) term.

**Figure 1:**
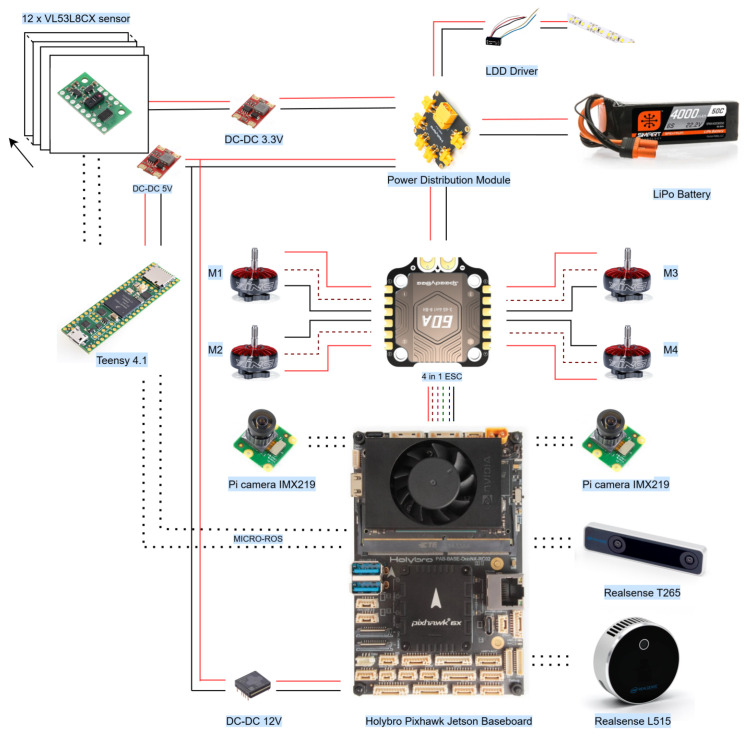
Comprehensive system architecture with all the components used in a UAV. It includes all the electronic components that suffice the need for autonomous navigation inside the ballast tank. However, the current study only focuses on the VL53L8CX sensor.

**Figure 2:**
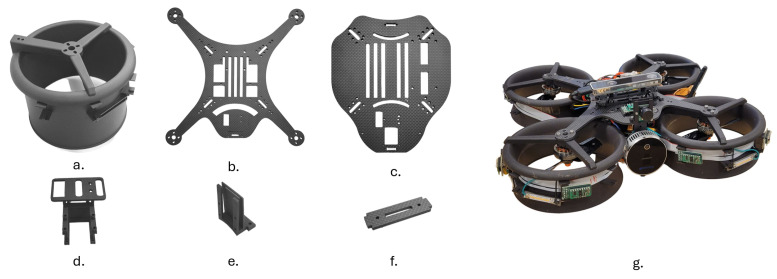
Airframe components of the quadcopter: (**a**) SLS-printed duct housing the time-of-flight sensors with motor; (**b**) upper carbon plate, attached to the duct at 5° and to the lower carbon plate (**c**) via supports (**f**); (**d**) platform for the lower Raspberry Pi camera module and VL53L8CX sensor; (**e**) enclosure for the front-facing Raspberry Pi camera; (**g**) complete assembly of all the components.

As depicted in Figure 1, the quadcopter features a Holybro Jetson Pixhawk baseboard as a companion computer, facilitating easier installation and providing a built-in interface between the Pixhawk 6X Pro flight controller and the Jetson Orin NX. A Spektrum 6S 4000 mAh LiPo battery providing 24 V with a power distribution board from Holybro is used to power all the electronic components and sensors. The IFlight XING 2806.5 1800 KV motor is used with a 5-inch bullnose propeller to provide a sufficient and consistent lift-to-weight ratio of 2. These motors are controlled with a 4-in-1 Speedybee 60A ESC. Two DC-DC voltage regulators, one with a 5 V output and the other with a 12 V output, are used for the Teensy 4.1 and Jetson baseboard, respectively. The Teensy microcontroller is further connected to 12 VL53L8CX sensors with an insulated ribbon cable. To provide a controlled lighting environment, four LED strips from Lumitronix are used with an LDD-700 LW driver, which is directly controlled from the autopilot for better efficiency in terms of power consumption and glare. As a part of the complete setup, the RealSense T265 and L515 cameras, along with a couple of wide-angle Sony IMX219 sensor configured with a Raspberry Pi camera module are used. The RPi cameras pointing forward and down serve the purpose of vision-based navigation while passing through the manholes in ballast tanks. Generally, these manholes are present either vertically on a wall or on the floor to allow the passage from one ballast tank compartment to another one. Furthermore, a T265 camera is used to aid the localization of UAV with visual inertial odometry (VIO) [64,65]. Finally, an L515 camera is used for mapping the environment due to its broad spectrum of vision [8]. However, these cameras are not the primary focus of this paper. The sensing configuration proposed in this work is intended, in the long term, to reduce reliance on localization and mapping cameras in confined-space missions, where camera-based methods are often constrained by payload weight and degraded performance in dark or low-texture environments. A future direction is to integrate end-to-end reinforcement learning with ToF depth reconstruction and monocular visual odometry to enable robust perception under such conditions; however, developing and validating that learning-based pipeline is beyond the scope of this paper. The fully assembled quadcopter, comprising all structural and electronic components as shown in Figure 2, has a total mass of 2.1kg, including the L515 and the T265 cameras with masses of 96g and 53g, respectively.

### 2.2. ToF Multi-Zone Sensor

The VL53l8CX TOF, a multi-zone ranging sensor from STMicroelectronics, is used. It claims to achieve a millimeter accuracy based on an advanced vertical cavity surface-emitting laser (VCSEL), a single-photon avalanche diode (SPAD) array, physical infrared (IR) filters, and diffractive optical elements (DOEs) [66]. One of the notable characteristics of this sensor is that it can be configured for 8 × 8 and 4 × 4 measuring zones, with frame rates of 15 Hz and 60 Hz, respectively. Any ToF error or interference around the 940 nm wavelength triggers an error flag, which can be used alongside a validity matrix to filter out noise and invalid data. Based on STMicroelectronics’ characterization, within the first 2 m, the sensor maintains an accuracy of around ±15mm and beyond this distance, measurement error gradually increases, potentially reaching up to 11% of the actual range. Several sensor parameters can be customized programmatically, namely the integration time, resolution, operating frequency, and sharpening features. It can be operated in continuous ranging mode or autonomous ranging mode based on the requirement. Functionally, the VL53L8CX is well suited for spatial awareness applications, such as estimating an object or detecting gaps using measured distances and known FoV geometry. The carrier board used for this sensor in this paper is from Pololu robotics and electronics, which provides an interface for both I^2^C and SPI communication. This essentially transforms the VL53L8CX into a simple 3D LiDAR system, as it captures multiple distance measurements rather than just a single point (like a 1D LiDAR), producing a low-resolution depth map of the surrounding environment within its field of view. The main specifications of the sensors are summarized in Table 1. Additionally, a comparison of power consumption and weight, determining the flight time in hovering state, is presented with other potential candidate systems in Table 2. As depicted, the use of this low-powered perception system consisting of Teensy and VL53L8CX sensors provides a proper balance in terms of flight time and weight compared to other possible alternatives.

### 2.3. TOF Sensor Setup

**Figure 3:**
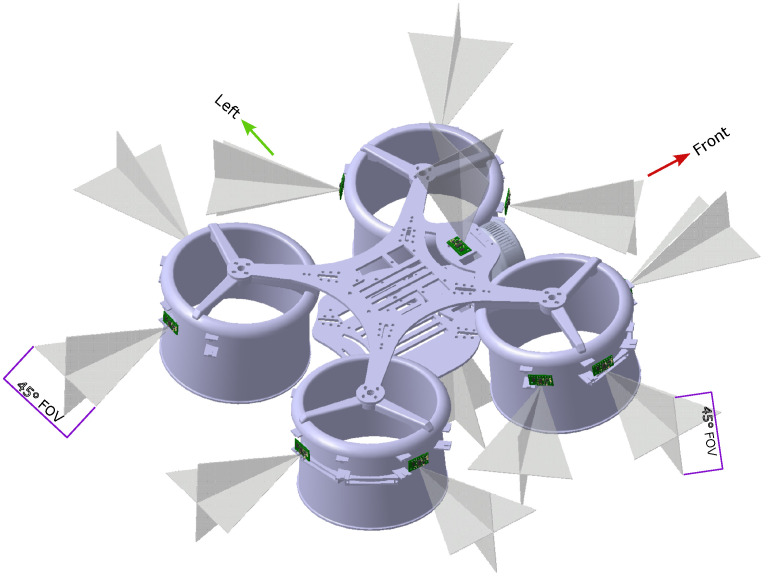
ToF sensors placement on the quadcopter for 360° circumferential coverage along with 45° vertical field of view.

The perception system of the quadcopter for the collision prevention consists of 12 VL53L8CX sensors around the quadcopter connected through a ribbon cable of length 1.1 m. The quadcopter has ten sensors placed on the circumference facing outwards, whereas one sensor is placed on the upper plate, facing upwards, and one is placed on the lower plate, facing downwards (12 ToF sensors in total), as shown in Figure 3. The sensors are arranged in such a way that the combined field of view covers a 360-degree circumference of the quadcopter, irrespective of symmetry in placement and without any individual FoV overlap.

**Figure 4:**
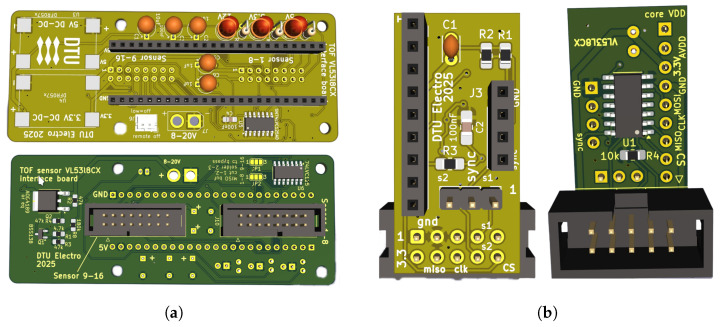
Interfacing module for Teensy 4.1 and 12 VL53L8CX sensors via ribbon. (**a**) Custom-designed PCB for microcontroller with two SPI buses. It houses slots for voltage regulators, buffers, transistors, and resistors for minimizing noise and interference; (**b**) custom-made PCB for VL53L8CX including buffer and synchronization circuit.

**Figure 5:**
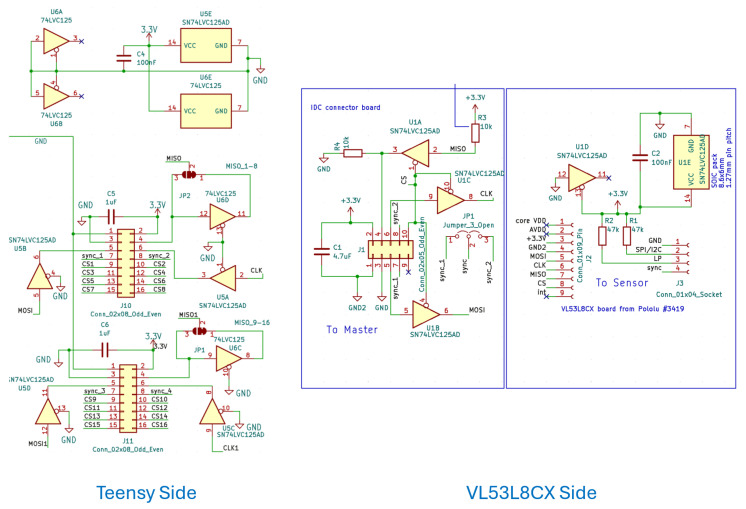
Circuit diagram for both the Teensy and VL53L8CX sensor sides, demonstrating the connection between different lines within the SPI buses.

The sensors are divided into 2 groups and connected to 2 SPI buses, wherein the first six sensors are connected to SPI1, which surrounds the front two ducts and upward. The other six sensors are connected to SPI2, which surrounds the rear two ducts and are downward. These buses are connected to a Teensy 4.1 microprocessor, which is selected for its RAM and processing speed. It has an ARM Cortex-M7 running at 600 MHz and is capable of handling data from all sensors with 1024 KB RAM. The individual sensor module interfaces with a dedicated custom PCB, shown in Figure 4, designed to mitigate noise during both initialization and data acquisition. A similar approach is used on the processor side, where the MISO, MOSI, and clock lines for each SPI bus are routed through external buffering and conditioning components (buffers, capacitors, and resistors) to strengthen signal integrity and ensure stable distance measurements. For each bus, two SN74LVC125A buffers (SOIC-14 package) are installed on the processor-side PCB. The MISO, MOSI, and clock signals are routed through the buffer channels referenced to ground (GND). On the sensor-side PCB, each sensor is connected through its own buffer, with the corresponding signal lines enabled by the sensor’s chip-select (CS) pin. Figure 5 shows a detailed connection of different components used in the sensor and Teensy side. This arrangement ensures that the Teensy 4.1 interacts with only one sensor per bus during initialization, avoiding bus contention.

**Figure 6:**
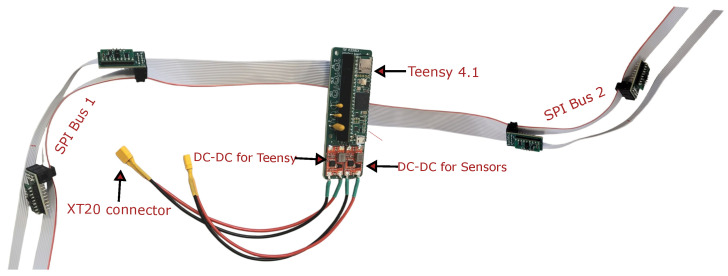
Physical representation of sensors connected to the Teensy 4.1 microprocessor through a ribbon cable along two SPI buses, one cable covering the front and another covering the rear part of the quadcopter.

The operating voltage for Teensy 4.1 is 5 V, and that of the VL53L8CX sensor is 3.3 V. For this, the PCB is also equipped with two step-down DC-DC converters, as shown in Figure 6, to maintain the operating voltage and power of the combined system through 24 V battery supply. The Teensy runs a micro ROS node to collect all the data from the sensors and send that to the Jetson as a topic.

**Figure 7:**
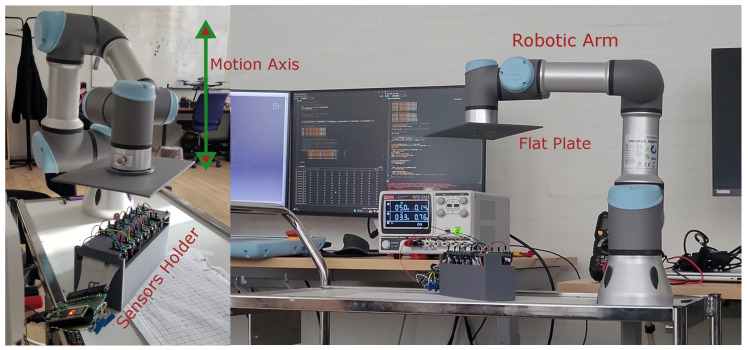
Experimental setup for the synchronization feature of VL53L8CX sensors. Twelve sensors are connected and attached in a test bed facing upward towards the moving obstacle plate attached to the robot arm.

12 ToF sensors are synchronized using a sync pin, which triggers all the sensors simultaneously during the reading phase. This is a significant characteristic for real-time perception and localization. This synchronization feature allows the sensors to read the distance around the quadcopter at the same instant of time rather than reading the distance sequentially. Sequential reading adds lag and can cause drift of the vehicle towards an unwanted direction. Several experiments were conducted with the help of a Universal Robot arm to verify this property, where all the sensors are connected and attached to a 3D printed custom holder facing upwards. The tool point of the robot arm is connected to a 3D printed flat plate, which is then moved vertically in a periodic motion such that it covers the FoV of all sensors at every iteration. Figure 7 shows the experimental setup employed to verify the synchronization property.

**Figure 8:**
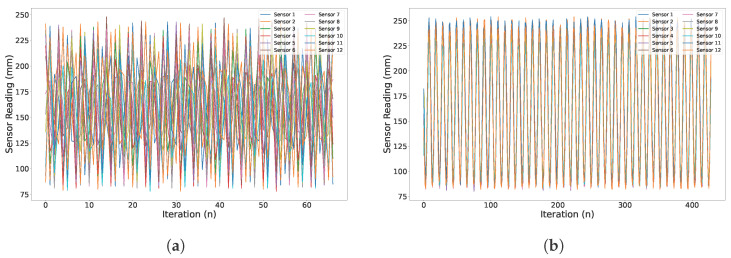
Dynamic distance measurement with and without the external trigger for synchronization. (**a**) Unsynchronized distance reading from the sensors, which is not useful for real-time control; (**b**) distance measurements are synchronized for all 12 sensors over time, replicating the motion of a moving platform.

From the plot in Figure 8b, it is clear that the readings from all sensors are almost identical at a given instant of time and position of the robot arm. The rise and fall of the peaks represent the position of the plate on the vertical axis. Each sensor is initialized with synchronization to be able to read the distance simultaneously from all sensors over a measuring iteration. In contrast, in Figure 8a, the readings are clearly not synchronised/overlapping since the sensors are measuring serially. This makes it clear that the synchronization feature is critical for real-time perception and control of the quadcopter. However, it is important to note that the frequency of the data acquisition decreases if the system is synchronized for a large number of sensors. For now, with 12 sensors, the maximum synchronized frequency that can be obtained is 12 Hz for 8×8 and 30 Hz for the 4×4 configuration. Furthermore, the resistance and capacitance of the respective resistors and capacitors need to be changed to incorporate more sensors in the system due to induced noise and interference. Hence, this does not make the system plug and play after the third sensor in each SPI bus in terms of scalability. Furthermore, due to the large number of sensors, the calibration of all sensors to perform as a single entity becomes a bit tedious.

**Figure 9:**
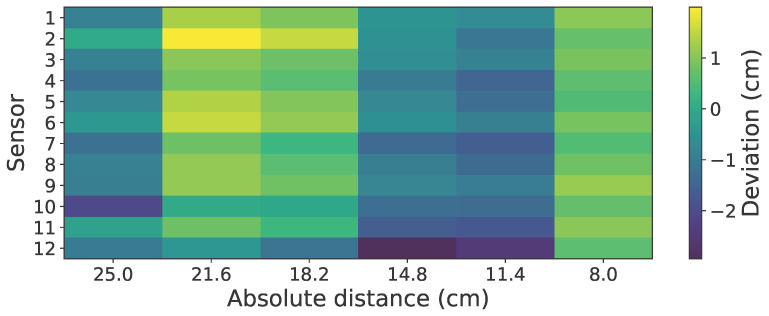
Mean deviation error heatmap across all sensors during the dynamic obstacle experiment, showing the consistency and reliability of distance reading from the sensor system.

The performance metrics for the accuracy are obtained from the same experiment as that of synchronization. The obstacle attached to the robotic arm is moved at a frequency of 1 Hz, covering the distance between 8 cm and 25 cm. Since the reading frequency of all the sensors is 12 Hz, we get an evenly spaced distance of 3.4 cm for each measurement instant along every half-cycle of the moving obstacle. From the distance readings obtained for each sensor, the absolute error per sensor is calculated, and a heat map of mean deviation across all strokes is generated as shown in Figure 9. The heat map reveals a structured trend: the error distribution is not random across the array, but changes systematically with target distance, indicating that the measurement bias depends on range rather than being dominated by timing jitter. Importantly, for a given instant, the 12 sensors report closely matched distances, and the deviation is highly repeatable across cycles, which supports the claim that the external triggering provides consistent sampling and that the sensing pipeline is stable. The observed range-dependent variation is likely attributable to optical effects such as target reflectivity and incidence angle. Overall, these results suggest that the system offers reliable and repeatable proximity measurements over the tested working range, while also highlighting the presence of a modest, range-dependent bias that could be further reduced through per-range calibration or compensation models if higher absolute accuracy is required.

**Figure 10:**
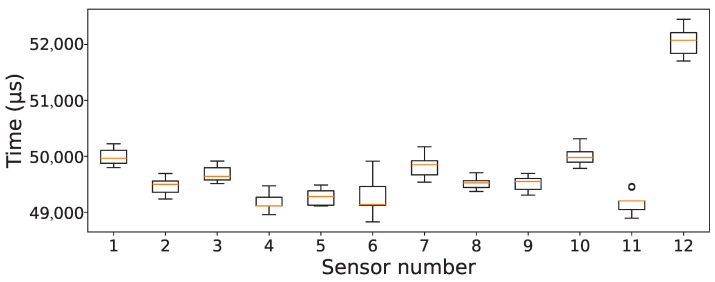
Per-sensor latency relative to synchronization trigger during the data ready and accumulation stage across both SPI buses.

Furthermore, the latency of the individual sensor is obtained and calculated during the flight. During this, different sensors were exposed to different obstacles at varying distances. Latency is measured as the time between the external SYNC trigger and the instant the microcontroller detects the availability of data from the sensor. Figure 10 shows the distribution of per-sensor latency during the synchronized distance ranging. Here, each box summarizes many trigger cycles for one sensor and reports the median, interquartile range, and outliers. The figure shows that the latency distributions are tightly clustered across sensors (around 50 ms), indicating consistent timing from triggering to data acquisition. This consistency is important for real-time collision prevention, as it implies that the fused obstacle representation is built from temporally aligned measurements with limited sensor-to-sensor skew. It can also be seen that sensor 12 exhibits a slightly longer acquisition time, which may be attributed to the increased physical separation between sensors 11 and 12.

## 3. Software-in-the-Loop (SITL) Simulation

This section discusses the architecture of the simulation environment for the designed framework showcased in the application of collision avoidance. SITL is capable of reproducing all scenarios in terms of vehicle dynamics and design, just like a realistic flight. This process is critical while validating new missions, control algorithms, or changing any important parameters before the flight. Hence, this work uses SITL for PX4 and interacts through Gazebo and QGroundControl.

### 3.1. PX4 SITL

PX4 firmware is structured into two primary layers: the flight stack, which manages core functionalities such as state estimation, control, navigation, and guidance; and the middleware, which handles internal and external communication. At the heart of the middleware is the micro Object Request Broker (uORB), a publish-subscribe messaging system that enables asynchronous data exchange between PX4 modules.

PX4 supports a software-in-the-loop (SITL) mode, allowing developers to simulate and test flight algorithms on a computer without physical hardware. The SITL architecture integrates two essential modules: the simulator and MAVLink. The simulator module interfaces with virtual environments such as Gazebo, while the MAVLink module manages communication with ground control stations (e.g., QGroundControl), offboard APIs, and companion computers.

**Figure 11:**
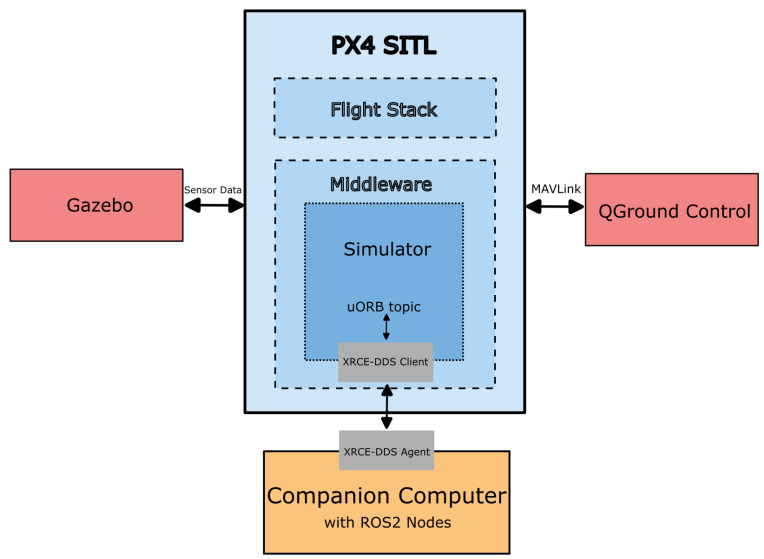
Block diagram with flow of signal between gazebo, ground control, and onboard computer in the PX4 SITL configuration used during the collision avoidance and prevention simulation.

Sensor data from the Gazebo simulator is transmitted via Gazebo plugins over predefined UDP ports. PX4 SITL listens on these ports, acquires the data through the Simulator module, and routes it via uORB for use by internal modules. Similarly, actuator commands generated by PX4 are sent back to Gazebo, closing the control loop. Ground control stations like QGroundControl connect to both PX4 and Gazebo through UDP, initiating handshake protocols to receive simulation data and provide mission control interfaces. This interconnected architecture, represented in Figure 11 enables seamless virtual testing of UAV behavior, sensor integration, and high-level control strategies in a realistic and reproducible environment.

### 3.2. PX4 SITL Gazebo Model

To evaluate the collision prevention capability of the quadcopter, a high-fidelity model is designed in CATIA V5 and integrated into the PX4 SITL-Gazebo simulation environment. Gazebo is a powerful open-source 3D simulator equipped with realistic physics engines and rendering tools, making it well suited for testing autonomous vehicle algorithms. It provides a modular framework that simulates rigid-body dynamics, visual and collision geometry, sensors, and environmental effects such as gravity, light, and wind.

The model is defined using the Simulation Description Format (SDF), which specifies the physical properties (mass, inertia), visual and collision geometries, sensor placements, and joint dynamics. Twelve VL53L8CX time-of-flight (ToF) sensors are positioned radially around the drone’s frame to provide omnidirectional distance measurements essential for collision detection and avoidance. These sensors are simulated using Gazebo sensor plugins for 3D LiDAR. This plugin is customized for the correct measurement range, update rate, FoV, and measuring zones, which publish range data over ROS2 topics for external processing.

Gazebo’s lock-step synchronization with PX4 SITL ensures deterministic message exchange between the simulator and PX4 firmware. The gazebo mavlink interface plugin relays sensor data and control messages between PX4 and Gazebo. The raw multi-array ToF sensor output is processed and transformed into suitable frames by a dedicated ROS2 node, which is then fed to a dedicated collision avoidance algorithm. As a part of this process, the raw readings obtained from the VL53L8CX time-of-flight (ToF) sensors are first organized into multi-arrays and then transformed into geometric frames for interpretation. Each sensor provides an 8×8 grid of distance values $d_{ij}$, where i,j∈{0,1,…,7}. Every element corresponds to a unique zone with an assigned ID, enabling the system to identify not only the measured distance, but also the exact position of the zone within the sensor’s field of view. These IDs are handy for defining customized sensing patterns depending on the operational requirements, such as emphasizing forward-facing zones for navigation or lateral zones for collision monitoring.

#### 3.2.1. Projection Model of the Sensor

**Figure 12:**
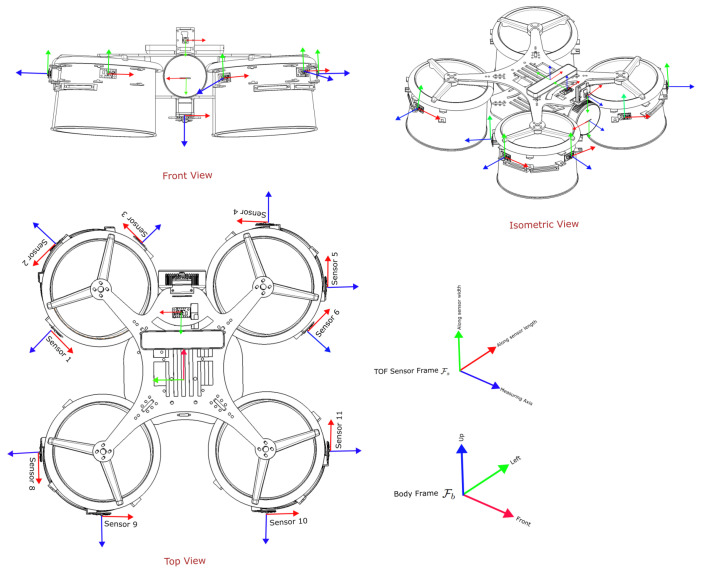
Wireframe diagram of the model showing placement and axes of the cameras and sensors relative to body frame $F_{b}$. The *z*-axis represents the measuring (optical) axis for all sensors.

Each zone measurement can be mapped to a ray in the local sensor frame $F_{s}$, represented in Figure 12. This is parameterized by the horizontal and vertical angular offsets $\left(\phi_{j},\theta_{i}\right)$. The VL53L8CX may thus be conceptually represented by a pinhole projection model, where all rays originate from a single optical center and diverge according to fixed angular steps. Using this model, the 3D coordinates of a point are derived as

(1) $$\begin{array}{cc} x_{ij} & =-d_{ij}\tan\left(\phi_{j}\right), \end{array}$$

(2) $$\begin{array}{cc} y_{ij} & =-d_{ij}\tan\left(\theta_{i}\right), \end{array}$$

(3) $$\begin{array}{cc} z_{ij} & =d_{ij}, \end{array}$$

where $z_{ij}$ lies along the optical axis and $\left(x_{ij},y_{ij}\right)$ represent lateral and vertical displacements. The negative signs account for axis alignment conventions in the sensor coordinate system.

#### 3.2.2. Rigid-Body Transformation to Base Frame

Each sensor is mounted at a fixed pose relative to the drone’s base frame $F_{b}$. A point expressed in $F_{s}$ can be written as

$$p_{F_{s}}=\left[\begin{array}{c} x_{ij}\\ y_{ij}\\ z_{ij} \end{array}\right]\in R^{3}.$$

To represent this point in $F_{b}$, a rigid-body transformation is applied using the extrinsic calibration between frames:

$$p_{F_{b}}=R_{F_{s}}^{F_{b}}\;p_{F_{s}}+t_{F_{s}}^{F_{b}},$$

where $R\in R^{3\times 3}$ is the rotation matrix and $t\in R^{3}$ is the translation vector. In homogeneous coordinates, this is expressed as

$$\left[\begin{array}{c} p_{F_{b}}\\ 1 \end{array}\right]=\underset{T_{F_{s}}^{F_{b}}}{\underset{︸}{\left[\begin{array}{cc} R_{F_{s}}^{F_{b}} & t_{F_{s}}^{F_{b}}\\ 0^{T} & 1 \end{array}\right]}}\left[\begin{array}{c} p_{F_{s}}\\ 1 \end{array}\right].$$

Not all raw measurements are suitable for use. To improve reliability, several filtering steps are applied as follows: (i) distances outside the valid operating range ($0.01<d_{ij}<3.5$ m) are discarded, (ii) readings caused by nearby floor or ceiling reflections are removed to prevent false lateral obstacle detections, and (iii) initialization errors are flagged when sensor returns deviate significantly from neighboring values. These criteria ensure that only physically consistent and meaningful measurements are retained. After filtering, the valid points from all *N* sensors are transformed into the standard base frame and combined to produce a single 3D pointcloud:

$$P=\overset{N}{\underset{k=1}{⋃}}\left\{p_{F_{b}}^{\left(k\right)}\;|\;\mathrm{valid}\left(p_{F_{s}}^{\left(k\right)}\right)\right\}.$$

This aggregated cloud provides a full 360° spatial representation around the drone and serves as input for downstream tasks such as mapping, navigation, and collision avoidance. In the ROS 2 framework, these fused measurements are typically published as PointCloud2 or equivalent depth messages relative to the body frame, enabling seamless integration with perception and planning modules.

**Figure 13:**
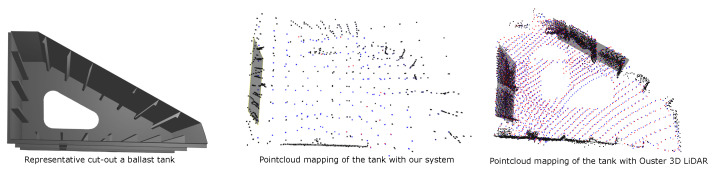
Comparison of the sparsity of pointcloud from the designed sensor setup with a commercially available 3D LiDAR while flying inside the mock-up ballast tank, whose model is shown in the first column.

To evaluate the spatial fidelity of the proposed multi-ToF perception system, side-by-side flights inside a ballast tank using our quadrotor and a commercial Flyability Elios3 equipped with a high-resolution rotating Ouster 3D LiDAR are conducted. In both trials, the vehicles were commanded to hover while performing slow yaw motions to collect range data under comparable conditions. From each data stream, ten representative frames were selected and visualized to assess the sparsity and reliability of the measurements during real flight rather than to benchmark full mapping performance. Figure 13 compares the resulting pointclouds and shows that, with strategic sensor placement, the fused VL53L8CX array produces a sparse but coherent pointcloud that is sufficient for spatial awareness and supports scan-matching-based pose refinement. While the lower point density mainly limits mapping fidelity, it does not compromise safety-critical perception, indicating that a lightweight, low-power multi-ToF configuration can serve as a practical alternative to a full 3D LiDAR in confined, metallic, and cluttered environments where size, weight, and cost constraints are critical.

### 3.3. Collision Avoidance

Sparse pointcloud data from all sensors is obtained, which is used to sense the vicinity and avoid any obstacles that may fall between the vehicle’s path and the target. A modified version of the vector field histogram (VFH+) [67] is used for the obstacle avoidance in this study. There are many recent studies based on this approach for obstacle avoidance with dynamic obstacles and cooperative path planning [68,69], which validate its consistency with LiDAR readings. However, the main objective of this paper is to verify the applicability of the designed perception system in terms of accuracy and reliability in confined spaces. This collision avoidance is implemented in PX4’s off-board control algorithm for multi-copter configurations.

All the valid points are flattened in xy coordinates and binned into 72 five-degree sectors around the quadcopter. For each bin, if at least two points are present, the minimum distance among those points is reported as an obstacle; otherwise, the bin is marked free by assigning the maximum range. Then, these 72 range values are published as a laser scan message. The message comprises an array of distance measurements covering the UAV’s surroundings. Each element in the array corresponds to a specific direction with distance measurement in millimeters (distances (72)), angular width in degrees between each measurement (increment), minimum and maximum distance (min_distance, max_distance), and orientation offset of the first measurement (angle_min).

**Figure 14:**
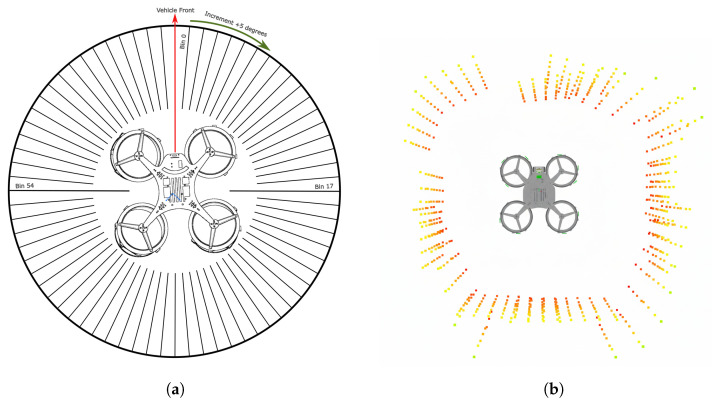
Visual illustration of obtained distance measurements from VL53L8CX sensors to be binned into sectors for collision avoidance. (**a**) Representation of binning of the surroundings as part of the sector division for collision avoidance around the quadcopter; (**b**) sparsity of points from all circumferential sensors while flying in a confined square area of 1m×1m in RViz2.

These parameters are better visualized with the figure Figure 14, which provides a 2D representation of the surrounding environment, divided into discrete angular segments—called bins—to interpret the spatial distribution of the obstacle.

This information is then used to command the quadcopter to navigate around obstacles towards a predefined target. The quadcopter moves straight towards the target until it finds an obstacle. Consequently, it builds a 72-sector polar histogram from the same ranges array, applies hysteresis and a safety margin around detected obstacles, identifies valleys of free space, and selects the candidate sector whose center has the minor absolute deviation from the goal direction. That sector is then smoothed and converted into an ENU heading, which is finally translated into a PX4-compatible NED yaw command. In this way, the algorithm uses the fused obstacle profile 360° to quickly choose a direct, unobstructed, or minimally deviated path around the obstacles.

The VFH+ algorithm used is based on [67], which follows the standard procedure, with some modifications according to the quadcopter used. This paper employs an eight-stage yaw angle computation, which is converted into a velocity setpoint in that direction and is fed to the PX4 controller to direct the vehicle towards the goal. The steps are briefly mentioned as part of the pseudo-code below (Algorithm 1).
**Algorithm 1:** Obstacle-Aware Sector Selection Using VFH+
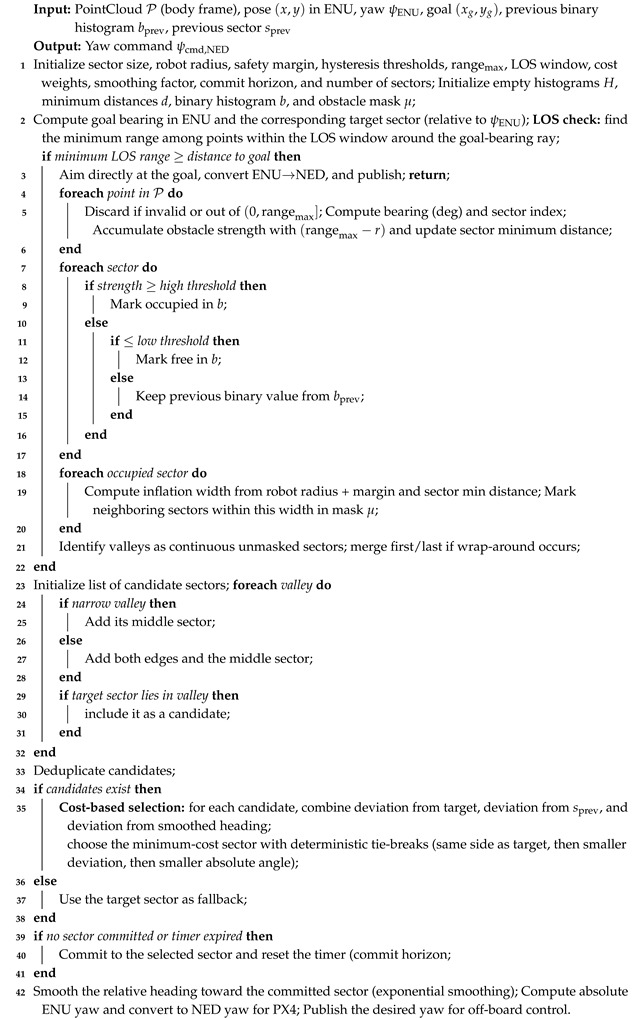


## 4. Simulation and Experimental Validation

The algorithm for collision prevention was first simulated in the Gazebo world with walls and obstacles, representing a very cluttered environment. The designed model was imported with all the sensors and dynamics as per the real-world application. A target was defined in an off-board control node and passed to the flight controller, such that the robot has to cross through obstacles during the flight. Real-time obstacle distance and minimum distance with desired yaw were calculated. This yaw, maintaining constant speed, was then converted to the desired velocity vector and used as the motion direction.

**Figure 15:**
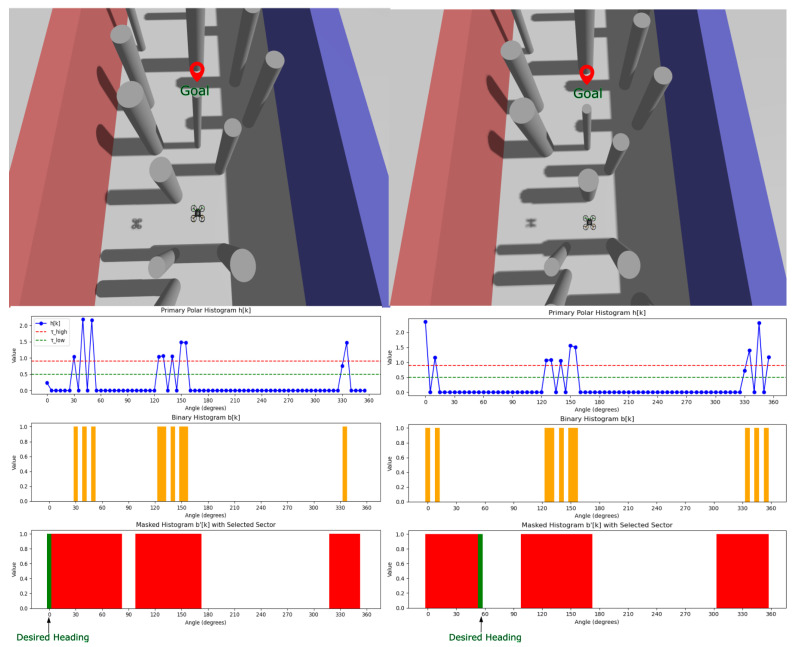
Visualization of the VFH+ algorithm while flying in a cluttered space; shows two different cases on how the quadcopter perceives its surroundings, creates different histograms, and finalizes the desired heading.

**Figure 16:**
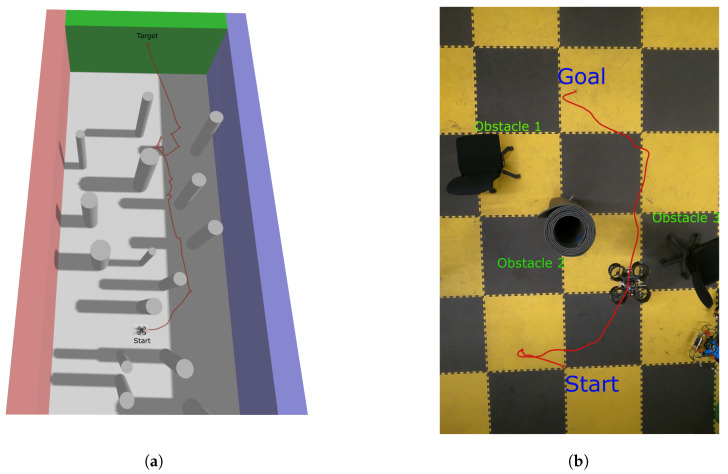
Validation of the designed perception system for collision avoidance in both simulated and real scenarios. (**a**) Path followed by the quadcopter running VFH+ algorithm for collision avoidance in a simulated cluttered space; (**b**) experimental setup with odometry of the quadcopter running the VFH+ algorithm for collision avoidance in a real flight.

Figure 15 depicts a real-time visualization of the VFH+ algorithm. It presents two different scenarios for obstacle placement, accompanied by a representation of the algorithm’s sectors. It provides a clear indication of how the system is perceiving its surroundings and creating histograms in different phases. In the first stage, range measurements are evaluated against a distance threshold (set to 1m) to focus on obstacles that are relevant for imminent collision risk. This is followed by the construction of a binary histogram that suppresses sectors associated with distant or irrelevant returns and retains only occupied directions. To further ensure safe clearance, the occupied sectors are then expanded by an additional 0.5m safety margin that accounts for the quadrotor’s physical dimensions and potential tracking error. Finally, the desired heading is selected by jointly considering the goal direction, the width of the remaining free sectors in the masked histogram, and a smoothness criterion that discourages abrupt steering changes. Figure 16a shows a path followed by the quadcopter in a simulation to reach a target while avoiding all the obstacles in between.

The designed perception system is validated using the VFH+ algorithm for collision avoidance in real flight. To perform this test, the quadcopter was flown in off-board control mode that interacts with the output of the VFH+ planner node to steer itself through velocity command. The Opti-track Mocap system was used for the localization of the quadcopter, which was fused with the internal IMU of the flight controller using an extended Kalman filter (EKF). Due to the limited flying area within the Opti-Track field of view, the number and height of obstacles were reduced compared to those of the simulation. All other communication protocols and channels were the same as in the simulation environment. Obstacles with different geometries were used to demonstrate its robustness. The quadcopter was flown at a constant height of 1m, and its odometry data was recorded to visualize the path followed. Figure 16b shows an instance of the flight between different obstacles to reach a goal. The quadcopter first moves to the left and finds that obstacles 1 and 3 are very close to one another, which is below its masked histogram threshold. Then, it goes towards the right and heads to the goal, maintaining a minimum cost sector throughout the path.

**Figure 17:**
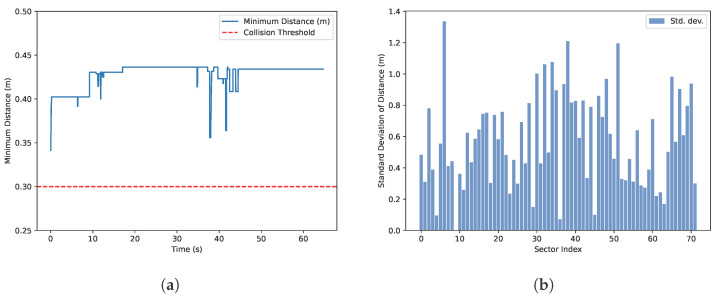
Graphical representation of matrices for the perception with the VL53L8CX sensor system during the real flight for collision avoidance. (**a**) Minimum distance measurement from all measuring zones around the quadcopter from takeoff to hovering at the destination; (**b**) standard deviation of the measured distances per sector throughout the flight.

Figure 17 visualizes the range measurements perceived by the circumferential sensor array during real flight. Throughout the trial, the quadcopter maintained a minimum clearance of approximately 0.45m from nearby structures, as summarized in Figure 17a. Because collision avoidance is activated only after the vehicle reaches its operating altitude, measurements recorded during the takeoff phase are excluded from the minimum-distance trace. As the quadcopter advances toward the goal while holding a fixed yaw orientation, the same subset of sectors continues to face the dominant nearby surfaces, which explains the extended intervals where the minimum distance remains nearly constant. In addition, Figure 17b reports the sector-wise variation in measured distance over the full flight duration. The larger deviations observed in specific sectors correspond to genuine changes in the surrounding geometry as the vehicle moves (e.g., encountering new obstacles or passing structural features), rather than instability in the sensor readings, indicating that the perception system responds consistently while capturing environmental transitions.

**Figure 18:**
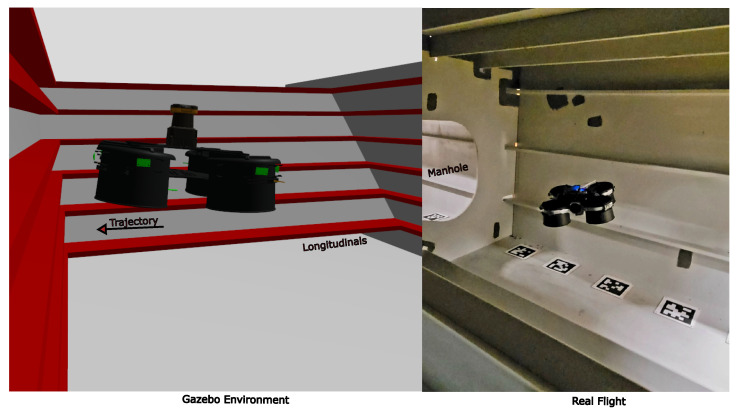
Collision prevention using the designed perception system in Gazebo and real ballast tank environment.

The proposed perception system is further evaluated for collision prevention during flight inside a mock-up ballast, tank Figure 18. Prior to the flight tests, a representative confined ballast-tank environment was recreated in Gazebo, incorporating common structural elements such as vertical longitudinals. This scenario differs from the planar-wall case because the protruding features can be difficult to detect reliably when only sparse range points are available. The experiment is conducted both in simulation and in real flight. In each test, the quadcopter is commanded to approach the wall at a constant velocity; once an obstacle is detected within 0.5 m, the controller generates a reverse thrust command and holds position. Because the collision-prevention algorithm bins measurements into angular sectors and selects the closest point in each sector, the quadcopter can reliably perceive wall protrusions. Consequently, the system does not require the sensor mounting plane to be aligned with the plane of the extruded longitudinal structures.

**Figure 19:**
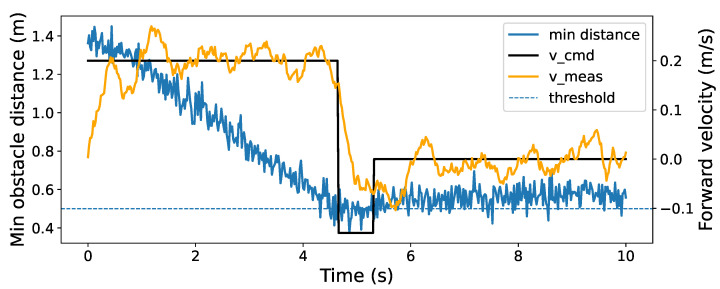
Collision prevention timeline representation of minimum distance and velocity during the flight inside the mock-up ballast tank.

Figure 19 illustrates the change in commanded (v_cmd) and responded (v_meas) velocity after takeoff, which is influenced by the minimum distance measured by the sensor system. As the quadcopter moves toward nearby structures, the minimum distance gradually decreases and acts as the primary trigger for the collision-prevention logic. When the proximity distance reaches the predefined safety condition, the controller transitions from the nominal forward motion to a reverse thrust command and subsequently enters a position-hold behavior to maintain a safe separation from the obstacle. The response velocity (v_meas) follows the commanded profile with a small delay, which is expected due to the vehicle dynamics and actuator response. It is worth noting that the reported minimum distance is computed exclusively from the circumferential array of sensors (i.e., the sensors providing horizontal coverage around the drone), while measurements from the sensors oriented toward the ceiling and the ground are excluded to avoid bias from irrelevant surfaces and to better reflect the lateral collision risk during the flight.

## 5. Conclusions

This paper presents the quadcopter design for confined spaces and the implementation of a novel perception system based on multiple VL53L8CX time-of-flight sensors. The physical and computational constraints of an aerial vehicle, including maneuverability, payload, and mission completion, are considered for agile flight. The proposed perception system is validated for its applicability in both simulation and real-world scenarios in confined spaces. This system leverages the multi-zone distance measuring capabilities for complete coverage of its surroundings, offering a promising lightweight alternative to conventional LiDAR-based perception methods. The designed system offers a good tradeoff between the weight, cost, and performance compared to 2D LiDAR (Hokuyo) and 3D LiDAR (Ouster). In addition to the reduction in cost, the flight time during hover is compared to the Ouster LiDAR configuration and it is increased significantly by almost 4min. Through a series of experiments and validation tests, we demonstrate the effectiveness and reliability of the developed sensor configuration for real-time collision prevention, obstacle avoidance, and feedback control applications. A modified vector field histogram is used for obstacle avoidance in both testing scenarios, which effectively navigates around different types of obstacles to reach the destination position. Additionally, future research will focus on integrating sensor fusion techniques with a lightweight visual system for optimal trajectory planning during ballast tank inspection.

## Figures and Tables

**Table 1:** Main specifications of VL53L8CX sensor.

Property	Value
Size	13 × 23 × 3 mm
Weight	0.7 g (without header pins)
Operating voltage	3.2–5.5 V
Max. range distance	4000 mm
Min. range distance	20 mm
Field of View (FoV)	65° diagonal; 45° horizontal/vertical
SPI speed	Upto 3 MHz
Price per piece	29.95 USD

**Table 2:** Comparison with the potential candidate systems.

Parameter	Teensy 4.1	12 × VL53L8CX	Hokuyo URG-04LX (2D LiDAR)	Ouster (3D LiDAR)
Operating voltage (V)	4.9	3.2	5	12
Operating current (A)	0.07	0.88	0.5	1.5
Power consumed (W)	0.343	2.816	2.5	18
Flight time (min)	7.8		8.1	4
Weight (g)	140		160	1100
Approx. Cost (USD)	400		1000	5000

## Data Availability

The relevant data and code can be found in https://github.com/basnet-susan/Osprey-TOF-system.git (accessed on 11 December 2025).
